# Migraine Features in Patients With Sudden Sensorineural Hearing Loss

**DOI:** 10.1002/ohn.70195

**Published:** 2026-03-09

**Authors:** Yalda Yazdani, Ella J. Lee, Amanda Francis, Saharnaz Nedjat, Mehdi Abouzari, Hamid R. Djalilian

**Affiliations:** ^1^ Department of Otolaryngology–Head and Neck Surgery University of California Irvine USA; ^2^ Department of Neurological Surgery University of California Irvine USA; ^3^ Department of Biomedical Engineering University of California Irvine USA

**Keywords:** cochlear migraine, headache, migraine, sudden sensorineural hearing loss

## Abstract

**Objective:**

To investigate the prevalence of migraine features among patients with sudden sensorineural hearing loss (SSNHL) and evaluate potential clinical associations.

**Study Design:**

Retrospective survey‐based study.

**Setting:**

Single institution tertiary care center.

**Methods:**

168 adult patients with SSNHL were recruited. Migraine diagnosis was determined using International Classification of Headache Disorders, 3rd edition (ICHD‐3) criteria. Patient demographics, migraine‐related features, and SSNHL characteristics were analyzed using univariate and multivariate logistic regression.

**Results:**

Of 168 SSNHL patients, 77 (46%) met full ICHD‐3 migraine criteria, which is higher than the general population. An additional 23% met the majority of the migraine criteria. Multivariate analysis revealed that aural fullness preceding hearing loss (*P* < .001), concordant laterality of hearing loss and headache (*P* < .001), hyperacusis (*P* = .006), otalgia (*P* = .01), and motion sickness (*P* = .03) were independently associated with migraine. Interestingly, 89% of patients with migraine headache and SSNHL had their SSNHL on the same side as their headaches.

**Conclusions:**

These findings reveal a high prevalence of migraine headache among patients with SSNHL. Clinical features, including aural fullness and concordant laterality (dominant headaches on the same side as SSNHL), were predictive of migraine in SSNHL patients. These findings support the hypothesis that migraine and SSNHL may share overlapping vascular and neurogenic mechanisms, highlighting the importance of identifying migraine features in SSNHL to optimize management and explore potential therapeutic strategies.

Sudden sensorineural hearing loss (SSNHL) is usually defined as an unexplained hearing loss greater than 30 decibels (dB) in three consecutive audiometric frequencies over 72 hours.^1^, ^2^ Hearing impairment affects men and women equally, and is mainly unilateral,^3^ with bilateral involvement in less than 5% of cases.^2^ Although incidence reports vary year by year and depending on the studied population, the incidence of SSNHL appears to increase as the population ages from children to older adults.^2^ Contemporary estimates indicate about 5 to 27 cases of SSNHL per 100,000 people in the United States.^2^, ^4^ However, these estimates are likely low as many patients with mild symptoms or rapid recovery might not seek medical care. There seems to be a slight male dominance, especially in patients aged 65 and older.^2^ Approximately 60,000 to 65,000 new cases of SSNHL are reported annually in the United States.^2^

It has been reported that SSNHL has no identifiable cause in over 90% of cases.^1^, ^5^, ^6^ Possible etiologies include infections, inflammation, head trauma, autoimmune disease, ototoxic drugs, attenuated blood flow or perfusion, neurological disorders, inner, and neoplasms along the cochleovestibular nerve.^1^, ^5^, ^6^, ^7^ SSNHL is considered an otologic emergency, requiring immediate recognition and treatment.^8^ High‐dose systemic corticosteroids should be administered immediately, as the most notable improvement typically occurs within the first 2 weeks, and intratympanic (IT) injections may be used as concurrent or adjuvant therapy.^9^

Migraine is a primary headache disorder and among the most common neurological conditions worldwide.^10^ Migraine headaches (MHs) are common and are estimated to affect about 18% of women and 6% of men worldwide.^11^ MHs have been associated with various otologic conditions, including recurrent benign paroxysmal positional vertigo (BPPV), aural fullness, persistent postural‐perceptual dizziness (PPPD), and recently SSNHL.^12^, ^13^, ^14^ An increasing amount of evidence indicates a link between migraine and SSNHL. Epidemiological studies have found about a 1.8‐fold higher incidence of idiopathic SSNHL among migraine sufferers compared to matched non‐migraine controls.^15^

On the other hand, patients with SSNHL tend to have a history of migraine more often than chance would predict, indicating a notable link between the two disorders.^16^ In one clinical series, nearly 40% of idiopathic SSNHL patients were also found to meet diagnostic criteria for migraine, a prevalence significantly higher than in the general population.^16^ In addition, a national database study of approximately 13,000 US adults revealed that migraine was independently associated with subjective hearing loss (25.0% vs 16.6%) and tinnitus (34.6% vs 16.9%) compared to individuals without migraine.^17^ Several hypotheses have been suggested regarding the pathophysiology of idiopathic SSNHL, highlighting its complex and multifactorial nature, yet a definitive mechanism remains unidentified.

In this study, we compare the prevalence of migraine features in a cohort of patients with SSNHL who meet the International Classification of Headache Disorders (ICHD‐3) criteria for MH to a cohort of patients with SSNHL who do not meet the criteria for MH to determine whether there is a difference between the cohorts.

## Materials and Methods

Following Institutional Review Board approval from the University of California, Irvine, surveys were administered to patients who presented to a tertiary care neurotology clinic with a chief complaint of sudden hearing loss during the past 3 years. All patients underwent audiometric testing, physical examination, and detailed history at the time of presentation. SSNHL was diagnosed prior to survey administration based on audiometric confirmation of a hearing loss of more than 30 dB across at least 3 consecutive frequencies occurring within 72 hours. Patients with conductive hearing loss, Ménière disease, fluctuating hearing loss, vestibular schwannoma, chronic otologic disease, or other identifiable causes of auditory symptoms were excluded during routine clinical evaluation. In addition, atherosclerotic vascular disease, metabolic disorders, autoimmune conditions, and medication use were ruled out by the as the cause of the hearing loss in this cohort. All patients were deemed idiopathic SSNHL.

The survey included questions assessing MH criteria adapted from the ICHD‐3 criteria (Table 1) and questions addressing SSNHL characteristics. Completed surveys were uploaded into a secure clinical database (REDCap; Vanderbilt University). MH classification was determined based on responses to the survey questions aligned with ICHD‐3 criteria and was confirmed by clinical history and assessment. The complete survey instrument is publicly available through the University of California, Irvine REDCap platform.

**Table 1:** Diagnostic Criteria for Migraine Headache Without Aura as Defined by the International Classification of Headache Disorders, 3rd Edition (ICHD‐3)

Diagnostic criteria o f migraine without aura
A. At least five attacks fulfilling criteria B to D
B. Headache attacks lasting 4‐72 hours (when untreated or unsuccessfully treated)
C. Headache has at least two of the following four characteristics:
1. unilateral location
2. pulsating quality
3. moderate or severe pain intensity
4. aggravation by or causing avoidance of routine physical activity (eg, walking or climbing stairs)
D. During headache at least one of the following:
1. nausea and/or vomiting
2. photophobia and phonophobia
E. Not better accounted for by another ICHD‐3 diagnosis
Diagnostic criteria of migraine with aura:
A. At least two attacks fulfilling criteria B and C
B. One or more of the following fully reversible aura symptoms:
1. Visual
2. Sensory
3. Speech and/or language
4. Motor
5. Brainstem
6. Retinal
C. At least two of the following four characteristics:
1. At least one aura symptom spreads gradually over ≥5 minutes, and/or two or more symptoms occur in succession
2. Each individual aura symptom last 5‐60 minutes
3. At least one aura symptom is unilateral
4. The aura is accompanied, or followed within 60 minutes, by headache
D. Not better accounted for by another ICHD‐3 diagnosis, and transient ischemic attack has been excluded.

Initial statistical analysis was performed between subgroups using univariate analysis. Second, multivariate analysis was performed on *P *< .05 using binary logistic regression to account for potential confounders. Statistical analyses were conducted using R (version 4.1.0; R Foundation for Statistical Computing) with built‐in functions from the default stats package. No external R packages were used.

## Results

**Figure 1:**
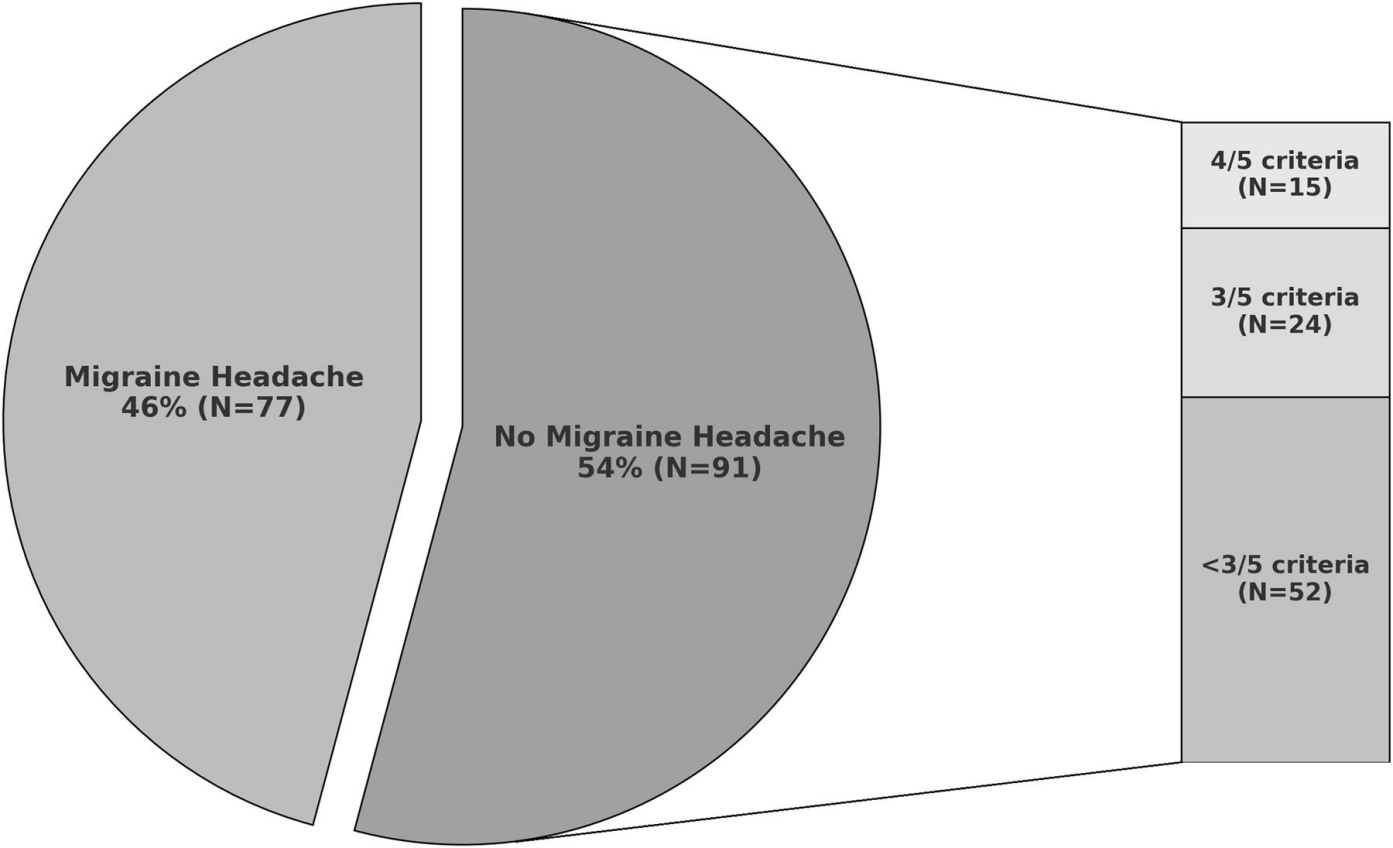
Percentage of patients with sudden sensorineural hearing loss who met ICHD‐3 criteria for migraine headache. ICHD‐3 indicates International Classification of Headache Disorders, 3rd edition.

A total of 168 patients with SSNHL and a history of headache were included in the study. The mean age of the patients was 55 ± 12 years. A total of 128 (76%) patients were female, and 40 (24%) were male. 86 (51%) patients had the onset of their hearing loss during a headache. 29 (17%) patients had a family history of migraine. A total of 77 (46%) patients met all 5 ICHD‐3 criteria (A‐E) for MH (Figure 1). Among patients who did not meet full criteria, 15 patients (16.5%) met 4 of the 5 criteria, and 24 patients (26%) met 3. The 15 patients who met 4 out of 5 migraine criteria were only missing 1 additional headache episode to fulfill the diagnostic criteria for migraine. Adding these patients who had experienced MHs but did not meet the number of headache attacks required by ICHD‐3 would make the MH group 55% of the SSNHL patients' cohort. Together, 69% of patients with SSNHL met all or a majority of the criteria for MH.

There were no notable differences in baseline characteristics between SSNHL patients who fulfilled the ICHD‐3 criteria for MH and those who did not (Table 2). Univariate analysis identified several clinical features that significantly differed between migraine and non‐migraine groups. These included concordant laterality of hearing loss and headache, aural fullness preceding headache, otalgia, sensitivity to sounds before the onset of SSNHL (hyperacusis), and motion sickness.

**Table 2:** Comparison of Basic Characteristics and Migraine‐Related Features in Survey Responders With Sudden Hearing Loss

Feature	MH cohort (N = 77)	Non‐MH cohort (N = 91)	*P* value
Age	55.4 ± 13.5	54.4 ± 10.5	.6
Sex			.6
Female	61 (79%)	67 (73%)	
Male	16 (21%)	24 (27%)	
Race			.9
White	42 (55%)	51 (56%)	
African American	10 (13%)	11 (12%)	
Asian	13 (17%)	15 (16%)	
Others	12 (15%)	14 (16%)	
Ethnicity Hispanic	15 (27%)	18 (20%)	.8
Motion sickness	45 (58.4%)	33 (36.3%)	.03*
Head/brain fog	39 (51%)	34 (37%)	.1
Aural fullness before HL	57 (74%)	20 (22%)	<.001*
Concordant Laterality of HL and Headache	63 (89%)	20 (22%)	<.001*
Neck stiffness	39 (51%)	33 (36%)	.08
Otalgia	33 (43%)	22 (24%)	.01*
Recurrent sinus headaches	25 (32.5%)	23 (25.4%)	.3
Ice cream headaches (brain freeze)	33 (43%)	36 (39%)	.6
Family history of migraine	13 (17%)	12 (13%)	.5
Family history of Meniere's disease	0 (0%)	3 (3.3%)	.3
Hyperacusis	24 (32%)	13 (14%)	.006*

Abbreviation: MH, migraine headache.

* Denotes a significant *P* value.

In multivariate logistic regression, all 5 mentioned variables remained statistically significant. The strongest associations were observed for aural fullness preceding the headache and concordant laterality of hearing loss and headache (*P* < .001). Specifically, 89% of patients with MH and SSNHL had their dominant headaches on the same side as the SSNHL. Hyperacusis before the onset of SSNHL (*P* = .006), otalgia (*P* = .01), and motion sickness (*P* = .03) were also independently associated with migraine diagnosis.

## Discussion

This retrospective study of 168 SSNHL patients demonstrated a significant association between SSNHL and MH. In our cohort, 77 (46%) patients met the ICHD‐3 migraine criteria, while the prevalence of MH in the general population is 15.3%.^11^ There were no significant differences in baseline demographics between migraine and non‐migraine groups. Our study supports the recent studies that have suggested a possible link between migraine and SSNHL.^17^, ^18^, ^19^, ^20^

Several population studies have found a link between general and SSNHL and migraine. A study of approximately 13,000 US adults demonstrated that migraineurs were more likely to have subjective hearing loss (25.0% vs 16.6%, *P* < .001) and tinnitus (34.6% vs 16.9%, *P* < .001) compared to the nonmigraineurs.^17^ Other extensive database analyses have also found that having a history of migraine increases the likelihood of developing sudden HL by 35% to 80%.^18^, ^19^, ^20^ Finally, treating patients with SSNHL using migraine prophylactic medications along with standard oral and intratympanic steroids has been shown to improve lower frequency hearing recovery.^6^ There appears to be evidence at the population level to link migraine and SSNHL and using adjuvant migraine medications with standard treatment improves outcome.

The underlying pathophysiology of SSNHL remains unknown, but several hypotheses have been proposed. Notably, many of these mechanisms overlap with those involved in migraine, indicating a possible shared cause. The most widely accepted theories involve vascular compromise and rupture of the cochlear membrane.^1^, ^5^ Ballesteros et al suggested a vascular etiology for idiopathic SSNHL as the blood supply to the cochlea arises from small terminal arteries.^21^ Unlike many organs with overlapping or redundant blood supply, the cochlea has no collateral circulation. The cochlear arteries, a branch of the labyrinthine artery, are the only source of arterial blood to the cochlea.^22^ There is no backup if this supply decreases or interrupted.^22^ The cochlear hair cells have a high metabolic demand and poor tolerance to hypoxia; even brief ischemia can cause transient or permanent hearing loss.^23^ Additionally, it is mentioned in several studies that vasospasm and vasodilation is commonly associated with migraine and may be the cause of certain symptoms such as monocular visual loss, aura, paresthesia, and vertigo.^24^, ^25^ In 1996, it was hypothesized that idiopathic hearing loss in pre‐existing migraine may be related to migraine‐induced vascular flow changes of the cochlear vasculature.^26^ Multiple subsequent studies have supported the idea that migraine may contribute to SSNHL through vasospasm or potentially vasodilation of the supplying arteries.^16^, ^27^ Together, these observations strengthen the theory that vascular dysfunction represents a shared mechanism linking migraine and SSNHL.

Another hypothesis for the development of SSNHL involves cortical spreading depression (CSD), which provides a neurogenic inflammatory link between migraine and SSNHL. In CSD, a wave of depolarization causes the release of neuropeptides such as calcitonin gene‐related peptide (CGRP) from the trigeminal nerve, leading to migraine aura and pain, along with auditory and balance issues.^28^, ^29^, ^30^, ^31^ Trigeminal nerve fibers have been shown to innervate the spiral modiolar artery and stria vascularis.^32^, ^33^ This has led to the hypothesis that trigeminal innervation acts as the link between migraine and associated cochleovestibular symptoms, including hearing loss, tinnitus, and vertigo.^32^ Experiments conducted in animal models have shown the presence of plasma extravasation in the cochlea due to alterations in vascular permeability after trigeminal nerve stimulation.^34^ These findings suggest that migraine‐related trigeminal activation may include cochlear dysfunction, providing a plausible mechanism for SSNHL.

Our study highlights specific features that may strengthen the association between migraine and SSNHL. Several clinical features were independently associated with migraine, with the strongest associations observed for aural fullness and concordance between the headache laterality and the side of the SSNHL (*P* < .001). To avoid overestimating the results, patients who reported headaches on both sides, regardless of migraine status, were classified as not concordant in our analysis. Migraine attacks are typically unilateral and often involve cortical spreading depression, activation of trigeminovascular system, and transient vasospasm with subsequent vasodilation.^15^, ^24^, ^25^ This could similarly compromise perfusion of the labyrinthine artery and cause ipsilateral hearing loss.^26^, ^27^ This vascular explanation is consistent with our findings, in which patients with MHs more often exhibited hearing loss on the same side as their headache, suggesting a shared lateralized pathophysiologic process. Likewise, aural fullness, a common audiovestibular symptom of migraine,^25^ may result from trigeminal activation and contraction of the tensor tympani muscle.^13^, ^35^ These clinical correlations reinforce a shared lateralized pathophysiology between migraine and SSNHL.

Other otologic symptoms further support this connection. Otalgia can occur as a referred migraine symptom via trigeminal convergence.^32^, ^33^ MH originates in the trigeminal nerve's meningeal afferents, and the trigeminal nerve also sends terminal sensory branches to critical structures within the cochlear vascular network.^32^, ^33^ Thus, activation of the trigeminal system during a MH can be perceived as otalgia. Consistent with this, a 2022 clinical study found that over 70% of patients with idiopathic otalgia met the criteria for migraine.^36^ These findings suggest that the migraine mechanism can cause referral otalgia, and treating the migraine in these patients led to improvement of the otalgia in 87% of cases.^36^ In addition, a retrospective review demonstrated that more than 90% of patients with chronic unexplained otalgia experienced significant relief with migraine medications.^37^

Beyond this, migraineurs are also reported to have a broad sensory hyperexcitability, which may explain why motion sickness and hyperacusis were overrepresented in our migraine patients.^38^ Several studies describe migraine as a disorder of altered brain excitability and sensory amplification.^38^, ^39^, ^40^ Migraineurs have a lower threshold for sensory input and exaggerated cortical response.^38^, ^39^, ^40^ Our team currently considers otologic migraine (migraine presenting with otologic symptoms) as part of the spectrum of central sensitization syndrome. Migraine patients score higher on the Hyperacusis Questionnaire and exhibit abnormal auditory event‐related potentials, indicating heightened auditory cortex excitability.^41^ In line with this, our previous study found that hyperacusis patients treated with migraine prophylaxis therapy experienced significant improvement in sound tolerance, as reflected by increased loudness discomfort levels and reduced symptom severity scores.^42^ Similarly, vestibular hypersensitivity related to migraine pathophysiology impairs the brain's ability to filter or adapt to motion signals, leading to motion sickness.^43^, ^44^, ^45^ Overall, these data support a model in which migraine‐related sensory hyperexcitability presents as various otologic and vestibular symptoms.

Our findings further support the association between SSNHL and migraine and highlight the importance of identifying patients with concomitant migraine symptoms who would potentially benefit from migraine treatment. A retrospective study of SSNHL patients done by our group demonstrated that adding migraine medications to the treatment regimen improved recovery of low‐frequency hearing loss, and reduced the total number of IT injections.^6^ Our group also showed that IT steroid combined with migraine medications significantly improved speech recognition threshold and long‐term hearing outcomes in patients with chronic (>3 months) SSNHL.^46^ These results suggest that SSNHL may have an underlying vascular or neurogenic inflammatory pathophysiology like migraine.

Since migraine diagnosis in this study was based on ICHD‐3 criteria, which classify headache type, only patients with a history of headache were included as having a diagnosis of migraine. We acknowledge that migraine‐spectrum disorders may present with minimal or absent headache, and therefore, some patients with SSNHL and migraine‐related pathophysiology may not have been captured in this cohort. This inclusion criterion may influence the reported prevalence of migraine features and could lead to underestimation of migraine‐spectrum involvement in the broader SSNHL population. The findings should be interpreted within the context of SSNHL patients who report headache symptoms. This study has several limitations inherent to its retrospective design, as well as selection bias related to how neurotologists at our institution identified SSNHL cases. Furthermore, the survey‐based nature of our data introduces the possibility of recall bias, which may affect statistical results and calls for careful interpretation of the findings. These findings reflect a tertiary care population and may not be generalizable. Future prospective studies with larger groups of patients experiencing both migraine and SSNHL are necessary to better understand clinical features that could assist in guiding treatment.

## Conclusion

This study reveals that 69% of patients with SSNHL met the majority of criteria for migraine headache (MH). In the cohort with unilateral headaches, 89% had their SSNHL on the same side as their headache. There were significant differences in migraine features between patients in our cohort with SSNHL who met the ICHD‐3 MH criteria and those who did not. The high prevalence of MH among this cohort could suggest a possible pathologic association between SSNHL and MH. Some patients with SSNHL may be part of the spectrum of otologic migraine, where migraine manifests as otologic symptoms.

## Author Contributions

**Yalda Yazdani**: data collection, data analysis, interpretation of the data, drafting of the manuscript, and final approval of the version to be published; **Ella J. Lee**: data analysis, interpretation of the data, drafting of the manuscript, and final approval of the version to be published; **Amanda Francis**: data collection, drafting of the manuscript, and final approval of the version to be published; **Saharnaz Nedjat**: data analysis, interpretation of the data, drafting of the manuscript, and final approval of the version to be published; **Mehdi Abouzari**: study conception and design, study supervision, interpretation of the data, drafting of the manuscript, and final approval of the version to be published; **Hamid R. Djalilian**: study conception and design, study supervision, drafting of the manuscript, and final approval of the version to be published.

## Disclosures

### Competing interests

Hamid R. Djalilian is an advisor and holds equity in NeuroMed Care LLC, Elinava Technologies, and Cactus Medical LLC.

### Funding source

Mehdi Abouzari was supported by the National Center for Research Resources and the National Center for Advancing Translational Sciences, National Institutes of Health, through Grant TL1TR001415.
